# Macrophage BMPRIB mediates p38-dependent inflammatory reprogramming and chemotaxis during ventilator-induced lung injury

**DOI:** 10.3389/fimmu.2026.1947415

**Published:** 2026-10-06

**Authors:** Jing Wang, Qi Sun, Lijuan Li, Junmei Xu, Lin Yang

**Affiliations:** 1 Department of Anesthesiology, The Second Xiangya Hospital, Central South University, Changsha, Hunan, China; 2 Hunan Province Center for Clinical Anesthesia and Anesthesiology, Research Institute of Central South University, Changsha, Hunan, China

**Keywords:** bone morphogenetic protein receptor (BMPR), inflammation, macrophage, NF-κB signaling, p38 MAPK, ventilator-induced lung injury (VILI)

## Abstract

**Background:**

Ventilator-induced lung injury (VILI) is a major iatrogenic complication of mechanical ventilation, in which dysregulated inflammation, particularly macrophage-driven response, amplifies pulmonary damage. Although bone morphogenetic protein (BMP) signaling exhibits context-dependent effects in pulmonary disorders, whether specific BMP receptors (BMPRs) regulate macrophage function during VILI remains unknown.

**Methods:**

Mice subjected to mechanical ventilation were randomly divided into Control (intubation without ventilation), LVT (low tidal volume), and HVT (high tidal volume) groups. HVT-exposed mice received intratracheal administration of exogenous BMP4 or the BMP type I receptor antagonist LDN193189. Lung injury severity was assessed by ultrasound, macroscopic scoring, wet/dry ratio, and histopathology. Macrophage accumulation, M1/M2 polarization, interstitial-to-alveolar macrophage (IM/AM) ratio, and BMP receptor expression were evaluated. Transcriptomic profiling was performed to identify differentially expressed genes and enriched pathways. In vitro, gain- and loss-of-function experiments (overexpression and knockdown of BMPRIB) were conducted in RAW264.7 macrophages to assess cytokine/chemokine production, migration, and MAPK pathway activation. The p38 inhibitor SB202190 was used to probe downstream signaling.

**Results:**

Compared with Control and LVT groups, HVT induced time-dependent macrophage accumulation, skewed M1/M2 polarization, and a progressive elevation of the IM/AM ratio. Strikingly, HVT selectively upregulated BMPRIB in lung macrophages, while downregulating BMPRII and leaving BMPRIA unchanged. Intratracheal BMP4 exacerbated these effects, whereas LDN193189 reversed BMP4-induced macrophage accumulation, M1 polarization, IM/AM elevation, and suppressed activation of ERK, JNK, p38, and p65 signaling. Transcriptomic analysis revealed that BMP4 enriched MAPK and inflammation-related pathways, with LDN193189 co-treatment partially reversing this enrichment. In BMP4-stimulated RAW264.7 macrophages, BMPRIB overexpression enhanced, whereas BMPRIB knockdown attenuated, cytokine/chemokine production and migration. Pharmacological p38 inhibition (SB202190) abrogated BMP4-induced pro-inflammatory effects in macrophages.

**Conclusions:**

HVT-induced BMPRIB upregulation in lung macrophages allows BMP4 to activate BMPRIB-dependent p38/NF-κB p65 axis as a key mediator of macrophage-driven inflammation in VILI Targeting the BMPRIB-dependent p38/NF-κB axis may represent a novel therapeutic strategy.

## Introduction

Mechanical ventilation is an indispensable life-support intervention for critically ill patients and is also routinely applied during general anesthesia and perioperative management (1). However, excessive mechanical stress generated by invasive ventilation can induce ventilator-induced lung injury (VILI), which is characterized by alveolar structural disruption, impairment of the alveolar–capillary barrier, and excessive inflammatory activation, resembling key features of acute respiratory distress syndrome (ARDS) (2, 3). Although lung-protective ventilation strategies, such as reduced tidal volume, limitation of plateau pressure, and control of driving pressure have been widely implemented in clinical practice, these approaches cannot completely prevent the occurrence of VILI (4). Therefore, elucidating the cellular and molecular mechanisms underlying VILI remains essential for developing novel therapeutic approaches.

Pulmonary macrophages represent key components of the innate immunity system and display remarkable phenotypic plasticity and functional heterogeneity (5). Macrophages are broadly categorized into M1-like and M2-like subsets. M1-like macrophages producing abundant pro-inflammatory cytokines and contribute to alveolar barrier damage, whereas M2-like macrophages participate in inflammation resolution and tissue repair (6, 7). Importantly, macrophage polarization is not static but dynamically shifts in response to local microenvironmental cues and a timely transition from M1- to M2-dominant responses is considered essential for effective tissue recovery (8). In the meantime, pulmonary macrophages mainly consist of resident alveolar macrophages (AMs) and interstitial macrophages (IMs), which can be distinguished based on the surface marker CD11C expression using flow cytometry (9). Under pathological conditions, the massive recruitment of monocyte-derived macrophages (MDMs)-which predominantly expand the IM pool-amplifies the M1 bias and impedes the necessary M2 switch (10, 11). Together, strategies aimed at regulating macrophage recruitment and restoring macrophage polarization balance may provide promising therapeutic opportunities for attenuating VILI.

As key members of transforming growth factor-β (TGF-β) superfamily, bone morphogenetic proteins (BMPs) play critical roles in embryonic lung development, tissue remodeling, and the pathogenesis of various respiratory diseases (12). Notably, accumulating evidence has revealed that BMPs exhibit context-dependent effects. A previous study has demonstrated that BMP2 and BMP4 exert distinct, and in some contexts opposing, effects in hypoxic pulmonary hypertension (13). This striking dichotomy is not fully understood; a plausible explanation lies in the differential expression and functional plasticity of BMPRs. Canonically, BMPs signal by forming receptor complexes in which type II receptors (BMPRII) bind the ligand and subsequently recruit type I receptors (primarily BMPRIA and BMPRIB), leading to activation of both SMAD-dependent and independent pathways (14). Multiple lines of evidence support this receptor-centric notion. For instance, BMPRIA and BMPRIB exert opposing effects on astrogliosis and inflammatory infiltration after spinal cord injury (15). Besides, BMPRIA promotes acute LPS-induced, but protects against chronic bleomycin-induced lung inflammation injury (16). All findings above indicate that distinct BMPR subtypes shall mediate divergent outcomes within the same pathological setting; and even the same receptor function may vary according to disease stage and inflammatory context. To date, the expression patterns of BMPRs and the mechanisms by which they regulate macrophage infiltration, phenotypic switching, and downstream signaling pathways in VILI remain largely unknown.

In this study, we hypothesized that VILI induces selective alterations in BMPR expression patterns, which subsequently regulate macrophage recruitment, phenotypic transition, and pulmonary injury. To test this, we established a mouse model of VILI by high tidal volume ventilation (HVT) and characterized BMPR expression profiles and cellular localization in injured lungs. Subsequently, exogenous BMP4 (a ligand agonist) and LDN193189 (a BMP type I receptor antagonist) were intratracheally administered to modulate BMP signaling activity. Furthermore, *in vitro* gain- and loss-of-function experiments were performed to dissect the downstream signaling pathways through which BMPRIB regulates macrophage function. Our findings may provide mechanistic insights into macrophage-mediated VILI, and identify potential therapeutic targets for intervention.

## Methods

### Animal

Specific pathogen-free (SPF) male C57BL/6 wild-type mice (7–9 weeks old; 20–25 g) were obtained from Hunan SJA Laboratory Animal Co., Ltd. (Changsha, China). Mice were housed under controlled conditions (22 ± 2 °C, 50 ± 10% humidity, 12-h light/dark cycle) with free access to food and water. Animals were acclimatized for at least 7 days before experimentation. All procedures were approved by the Animal Ethics Committee of the Second Xiangya Hospital of Central South University (Approval No. 20220278) and performed in accordance with the guidelines of the National Institutes of Health for the Care and Use of Laboratory Animals.

### Tracheal intubation

Tracheal intubation was performed as described previously (17). Briefly, mice were anesthetized with 1% sodium amobarbital (100 mg/kg, intraperitoneally) and placed supine on a surgical platform with limbs gently secured. After disinfection of the neck skin with 75% ethanol, the platform was tilted to 45°. A 22G intravenous catheter fitted with a custom-made light-guiding stylet was used to transilluminate the pharynx and visualize the glottis. The catheter was carefully inserted through the vocal cords into the trachea at the glottic opening and advanced to a depth of approximately 1.2 ± 0.5 cm. Correct placement was confirmed by symmetrical chest wall movement. The catheter was then connected to a small-animal ventilator (R415 VentStar, RWD Life Science, Shenzhen, China).

### VILI model validation and experimental design

To evaluate ventilation strategy-dependent lung injury, mice were randomly allocated into three experimental groups: ① Control group: intubated but not connected to the ventilator, maintained under anesthesia for 2 h; ② LVT group: intubated and ventilated with low tidal volume (8 mL/kg, 80 breaths/min, I:E ratio 1:1) for 2 h; ③ HVT group: intubated and ventilated with high tidal volume ventilation (30 mL/kg, 120 breaths/min, I:E ratio 1:1) for 2 h. Following ventilation, mice were deeply anesthetized with an intraperitoneal injection of sodium pentobarbital (300 mg/kg) and subsequently euthanized by cervical dislocation at four predetermined time points: 2 h after mechanical ventilation (P0), and at day 1 (P1), day 3 (P3), and day 7 (P7) after ventilation. Lung tissues were harvested for western blotting and RT-PCR analyses. Additionally, at P3, additional lung tissues were also collected for histological examination and flow cytometry analysis.

To investigate the role of BMP signaling in VILI, all mice were subjected to HVT for 2 h as described above, and randomly allocated to the following three groups: (1) HVT+PBS group: mice received sterile phosphate-buffered saline (PBS); (2) HVT+BMP4 group: mice received 200 ng exogenous recombinant mouse BMP4 (#315-27-01M, PeproTech, Rocky Hill, NJ, USA) dissolved in 40 μL sterile PBS; (3) HVT+B+L group: mice received 200 ng BMP4 combined with 10 μg LDN193189 (a specific BMPRI antagonist, S7507, Selleck, TX, USA) dissolved in 40 μL sterile PBS. Mice received intratracheal instillations daily for 3 consecutive days starting immediately after ventilation. After each instillation, they were returned to their cages and monitored. On P3 after the first instillation, all mice were euthanized following the same protocol described above, and lung tissues were collected for subsequent analyses.

### Cell culture and stimulation

The murine macrophage cell line RAW264.7 were purchased from Wellbiology Co., Ltd. (Changsha, China). Cells were cultured in Dulbecco’s Modified Eagle’s medium (DMEM; C11995500BT, Gibco, Thermo Fisher Scientific, Waltham, MA, USA) supplemented with 10% fetal bovine serum at 37 °C in a 5% CO_2_ incubator. When cells reached 50–60% confluence, transfections were performed using NanoTrans™ Transfection Reagent 3000 (CT0006, CYTOCH Biotech, Shanghai, China) according to the manufacturer’s instructions. For BMPRIB knockdown, cells were transfected with a specific siRNA targeting mouse BMPRIB (siBMPRIB, HX-M-S3-06245, Haixing Biology, Hangzhou, China) or a scrambled control siRNA. For BMPRIB overexpression, cells were transfected with pcDNA3.1-musBMPRIB-3xFlag (HG-M0007560, HonorGene, Changsha, China) or the empty pcDNA3.1 vector. At 6 h post-transfection, the medium was replaced with fresh complete DMEM, and transfection efficiency was confirmed 48 h later by RT-PCR and fluorescence staining.

To determine the optimal BMP4 concentration for subsequent experiments, RAW264.7 macrophages were treated with recombinant mouse BMP4 at concentrations of 0, 10, 50, and 100 ng/mL for 48 h. The mRNA levels of inflammatory and chemotactic genes were then examined by RT-PCR (**Supplementary Figure 1**). Stimulation with 100 ng/mL BMP4 significantly upregulated the expression of ICAM-1, CCL2, and IL-6 (each P<0.05, **Supplementary Figures 1A, E, F**), whereas CD16, CD86, CXCL2 and TNF-α showed no statistically significant changes (P>0.05, **Supplementary Figures 1B–D, G**, except for TNF-α, which showed upward trends but did not reach statistical significance, P = 0.06). Based on these results, 100 ng/mL BMP4 was selected for all subsequent *in vitro* experiments.

Three independent sets of experiments were performed. First, to determine the necessity of BMPRIB in mediating BMP4 effects, cells were transfected with siBMPRIB or scrambled control siRNA (si-SCR) for 48 h, and then stimulated with 100 ng/mL recombinant mouse BMP4 (#315-27-01M, PeproTech) for an additional 48 h. The following groups were included: (1) si-SCR (Control), (2) si-SCR+BMP4, and (3) si-IB+BMP4. Second, to assess whether BMPRIB overexpression is sufficient to enhance BMP4 responses, cells were transfected with the BMPRIB overexpression plasmid or empty pcDNA3.1 vector for 48 h, and then stimulated with 100 ng/mL BMP4 for an additional 48 h. The groups included: (1) Vector (Control), (2) Vector+BMP4, and (3) oe-IB+BMP4. Third, to verify the involvement of p38 MAPK as a downstream effector, cells were pretreated with 1 μmol/L SB202190 (SB, a specific p38 MAPK inhibitor, E5786, Selleck Chemicals, Houston, TX, USA) for 6 h, followed by stimulation with 100 ng/mL BMP4 for 48 h. The groups included: (1) control (no treatment), (2) BMP4, and (3) BMP4+SB. After the respective treatments, cells were collected for subsequent analyses.

### Lung ultrasound examination

Lung ultrasound was performed using a VINNO 6 LAB small animal ultrasound system (VINNO Technology, Suzhou, China) equipped with a 23-MHz linear transducer. Prior to examination, the thoracic hair of mice was removed to ensure optimal acoustic coupling. The transducer was placed directly on the chest wall of anesthetized mice, with the imaging depth standardized to approximately 1 cm. Representative ultrasound images were acquired to assess VILI-related features including B-lines, pleural irregularities, and interstitial syndrome.

### Lung wet/dry ratio

The right upper lobe of the mouse lung was dissected and immediately weighed to obtain the wet weight. Subsequently, the tissue was dried in an oven at 65 °C for 72 h, and the dry weight was recorded. The W/D Ratio was calculated as wet weight divided by dry weight.

### Histopathological analysis

Lung tissues were fixed in 3.7% paraformaldehyde, embedded in paraffin, and sectioned at 4-5 μm thickness. After deparaffinization, the sections were stained with H&E staining and examined under a light microscope. Lung injury was scored semi-quantitatively based on established criteria (18) for the following parameters: alveolar congestion, hemorrhage, inflammatory cell infiltration, and alveolar wall thickening. Each parameter was graded on a scale from 0 to 3 (0=no injury; 1=mild; 2=moderate; 3=severe), and the individual scores were summed to obtain a total lung injury score (range 0-12).

### Western blotting analysis

Cells and lung tissues were homogenized in RIPA lysis buffer (PC101, Epizyme, Shanghai, China) supplemented with protease inhibitor (GRF101, Epizyme) and phosphatase inhibitor (GRF102, Epizyme), and centrifuged at 4 °C at 12,000 rpm for 15 min to collect the supernatants. Protein concentrations were determined by the bicinchoninic acid (BCA) assay (500-0001, Bio-Rad, CA, USA) and measured with a microplate reader at 562 nm. Equal amounts of protein (20 μg per sample) were loaded onto SDS-polyacrylamide gels for electrophoresis, and transferred to polyvinylidene difluoride (PVDF) membranes (GVWP02500, Millipore, MA, USA). Membranes were blocked with 5% non-fat dried milk for 1 h at room temperature, and incubated overnight at 4 °C with primary antibodies including rabbit anti-BMPRIB (1:1000, ab155058, Abcam, Cambridge, UK), mouse anti-BMPRII (1:2000, 68497-1-Ig, Proteintech, Rosemont, IL, US), rabbit anti-BMPRIA (1:1000, ab264043, Abcam), rabbit anti-phospho-p38 (1:1000, #4511, Cell Signaling, Danvers, MA, US), rabbit anti-p38 (1:1000, #9212, Cell Signaling), rabbit anti-phospho-JNK (1:1000, #4668, Cell Signaling), rabbit anti-JNK (1:1000, #9252, Cell Signaling), rabbit anti-phospho-ERK (1:1000, #4370, Cell Signaling), rabbit anti-ERK (1:1000, #4695, Cell Signaling), rabbit anti-phospho-p65 (1:1000, #3033, Cell Signaling), rabbit anti-p65 (1:1000, #8242, Cell Signaling), VE-cadherin (1:1000, SC-52751, Santa Cruz, Dallas, TX, USA), PECAM-1 (1:1000, SC-20071, Santa Cruz), rabbit anti-CD86 (1:1000, NBP2-25208, NOVUS, Centennial, CO, USA), rabbit anti-CD204 (1:3000, 33649-1-AP, Proteintech), rabbit anti-CD11b (1:1000, ab52478, Abcam) and β-actin (1:10000, AF7018, Affinity, OH, USA). Membranes were then incubated with HRP-conjugated goat anti-rabbit IgG (SA00001-2, 1:6000, Proteintech) or HRP-conjugated goat anti-mouse IgG (SA00001-1, 1:6000, Proteintech) for 1 h at room temperature. Following three washes with TBST, signals were developed using ECL reagent (WBKLS0050, Millipore) and visualized with an imaging system (CLINX, Shanghai, China). Band intensities were analyzed with Image-Pro Plus 6.0 software (Rockville, MD, USA).

### Real time polymerase chain reaction

Total RNA was extracted from cells and lung tissues using TRIzol™ reagent (DP424, TIANGEN Biotech, Beijing, China). RNA purity and concentration were assessed by spectrophotometry. First-strand cDNA was synthesized from 1 μg of total RNA using the FastKing cDNA Reverse Transcription Pre-Mix Kit (KR126, TIANGEN). Quantitative real-time PCR was performed with the FastReal qPCR PreMix (SYBR Green; FP217, TIANGEN) on a real-time PCR Detection system (Bio-Rad Laboratories, Hercules, CA, USA).

The cycling conditions consisted of an initial denaturation at 95 °C for 3 min, followed by 40 cycles of 95 °C for 5 s and 50-60 °C (depending on the primer) for 30 s. Melt curve analysis was performed to confirm single-product amplification. GAPDH was used to normalize gene expression. The relative gene expressions were calculated using 2^-ΔΔCT^ method as previous introduced (19). The primer sequences were listed in **Table 1**.

**Table 1:** The primer sequences of target genes.

Gene	Forward primer (5′→3′)	Reverse primer (5′→3′)
β-actin	CCTCACTGTCCACCTTCC	GGGTGTAAAACGCAGCTC
CD86	TGTTTCCGTGGAGACGCAAG	TTGAGCCTTTGTAAATGGGCA
CD204	TGCTATCTCAGTGCAATCATCA	CGAGGAGGTAAAGGGCAATC
CD16	TTTGGACACCCAGATGTTTCAG	GTCTTCCTTGAGCACCTGGATC
IL-6	TAGTCCTTCCTACCCCAATTTCC	TTGGTCCTTAGCCACTCCTTC
IL-10	GCTCTTACTGACTGGCATGAG	CGCAGCTCTAGGAGCATGTG
YM-1	CTCTCCAGAAGCAATCCTGAAGAC	GCCCAACTGGTATAGTAGCACATC
CCL2	TTAAAAACCTGGATCGGAACCAA	ACCCGTAAATCTGAAGCTAATGC
ARG-1	CTCCAAGCCAAAGTCCTTAGAG	GGAGCTGTCATTAGGGACATCA
TNF-α	CCTGTAGCCCACGTCGTAG	GGGAGTAGACAAGGTACAACCC
ICAM-1	GTGATGCTCAGGTATCCATCCA	CACAGTTCTCAAAGCACAGCG
CXCL2	CACTCTCAAGGGCGGTCAAA	GCTCCTCCTTTCCAGGTCAGTTA
BMPRIB	CAGAAGTGCTGGACGAGAGCTT	GGTCGTGATAGGGAAGCTGGTA

### Flow cytometry

For analysis of lung macrophages, single-cell suspensions were prepared from lung tissues by digestion with Liberase™ (05401119001, Roche, Mannheim, Germany) and DNase I (11284932001, Roche) at 37 °C for 30 min. The digested mixture was filtered through 70 μm cell strainers, and residual tissues were gently ground with a 1 mL syringe plunger. Cell pellets were treated with red blood cell lysis buffer for 15 min in the dark and then centrifuged to remove impurities.

Cells were washed and resuspended in cold PBS supplemented with 2% bovine serum albumin (BSA) and ethylenediaminetetraacetic acid (EDTA). Cells were stained with Live/Dead BV510 dye for dead cell exclusion and incubated with Fc blocking reagent to inhibit non-specific antibody binding. Subsequently, cells were incubated with a cocktail of the following antibodies: APC-Cy7-anti-CD45 (557659, BD Pharmingen, San Jose, CA, USA), PerCP-Cy5.5-anti-GR-1 (552093, BD Pharmingen), FITC-anti-CD11b (557396, BD Pharmingen), PE-anti-F4/80 (565410, BD Pharmingen), PE-Cy7-anti-CD86 (560582, BD Pharmingen), BV421-anti-CD163 (155309, BioLegend, San Diego, CA, USA) and APC-anti-CD11c (550261, BD Pharmingen). Afterwards, cells were incubated at 4 °C for 30 min in the dark, washed, and resuspended in staining buffer.

Flow cytometric data were analyzed using FlowJo v10.8 software. Single-stained controls and fluorescence-minus-one (FMO) controls were applied for compensation and gating validation. The gating strategy was as follows: GR-1^+^CD11b^+^ neutrophils were excluded, and F4/80^+^CD11b^+^ cells were defined as lung macrophages. M1-like (CD86^+^CD163^-^) and M2-like (CD86^-^CD163^+^) macrophages were distinguished to calculate the M1/M2 ratio. Additionally, CD11c^+^ alveolar macrophages (AMs) and CD11c^-^ interstitial macrophages (IMs) were classified to evaluate the IM/AM ratio.

### Immunofluorescence

Immunofluorescence staining was performed to identify the cellular source of BMPRIB in mouse lung tissues. Mouse lung sections were fixed with 3.7% paraformaldehyde, permeabilized with 0.2% Triton X-100, and blocked with 5% bovine serum albumin (BSA) containing 10% normal goat serum to eliminate non-specific binding. The sections were then incubated overnight at 4 °C in a humidified chamber with primary antibodies including rabbit anti-BMPRIB (1:250, ab155058, Abcam) and rat anti-F4/80 (a macrophage marker, 1:200, #71299, Cell Signaling) or rat anti-CD31 (an endothelial cell marker, 1:50, ab256569, Abcam). After washing with PBS, the sections were incubated with a mixture of Alexa Fluor 488-conjugated Donkey Anti-Rabbit IgG (H+L) (1:100, 711-545-152, Jackson ImmunoResearch, West Grove, CA, USA) and Alexa Fluor 594-conjugated Donkey Anti-Rat IgG (H+L) (1:100, 712-585-150, Jackson ImmunoResearch) for 1 h at room temperature in the dark. Nuclei were counterstained with 4′,6-diamidino-2-phenylindole (DAPI). Images were acquired using a confocal laser scanning microscope, and co-localization analysis was performed to determine whether BMPRIB was expressed in macrophages (F4/80^+^) or endothelial cells (CD31^+^).

### RNA-sequencing

RNA-seq analysis was performed on lung tissue samples by Tianjin Tsingke Biotechnology Co., Ltd. (Tianjin, China). Total RNA was isolated using TRIzol reagent. RNA purity and integrity were evaluated with a NanoDrop 2000 spectrophotometer (Thermo Fisher Scientific, Waltham, MA, USA) and an Agilent 2100 Bioanalyzer (Agilent Technologies, Santa Clara, CA, USA). Sequencing libraries were prepared and sequenced on an Illumina NovaSeq 6000 platform. Raw reads were subjected to quality filtering to remove low-quality sequences, yielding clean reads. The clean reads were aligned to the mouse reference genome (GRCm38) using HISAT2 software, and gene expression levels were quantified as FPKM (fragments per kilobase of transcript per million mapped reads) using StringTie. Differentially expressed genes (DEGs) were screened based on the criteria of P < 0.05 and |fold change| > 2. Functional enrichment analyses, including Gene Ontology (GO) and Kyoto Encyclopedia of Genes and Genomes (KEGG) pathway analyses, were performed using the hypergeometric distribution test in R software (version 4.1.3) with RStudio (version 1.4.1717).

### Transwell migration assay

Sterile 24-well Transwell^®^ inserts with 3.0 μm pore polycarbonate membranes (3414, Corning, Corning, NY, USA) were used for the migration assay. Supernatants from BMP4-stimulated RAW264.7 cells were added to the lower chamber as chemoattractants. RAW264.7 cells were resuspended in serum-free medium at a density of 4 × 10^6^ cells/mL, and 100 μL of the cell suspension was seeded into the upper chamber. After 48 h of incubation at 37 °C in 5% CO_2_, non-migrated cells on the upper surface of the membrane were gently removed with a cotton swab. Migrated cells on the lower surface of the membrane were fixed with 4% paraformaldehyde and stained with 0.1% crystal violet. Cells were then photographed and counted in three random fields under an inverted microscope to assess cell migration capacity.

### Statistical analysis

Data of animal and cell studies were presented with the mean and standard deviation. For comparisons of airway plateau pressure between LVT and HVT groups, unpaired two-tailed Student’s t-test was used. For comparisons involving morphological changes, western blotting, RT-PCR, flow cytometry data and *in vitro* experiments, one-way ANOVA followed by Sidak’s multiple comparison test was applied. Correlation between BMPRIB expression and CD11b levels, and between p65 and MAPK signals phosphorylation were evaluated using Pearson’s correlation coefficient. A two-tailed P value < 0.05 was considered statistically significant.

All data were analyzed and graphically presented using SPSS (version 19.0, IBM, Armonk, NY, USA) and Prism software (version 10.0.1, GraphPad Inc., Boston, CA, USA).

## Results

### HVT induces acute lung injury

Airway plateau pressure was significantly higher in the HVT than in the LVT group (20.5 ± 3.6 vs. 10.8 ± 2.2 cm H_2_O, P<0.001, **Figure 1A**). Then, lung injury was evaluated by multiple parameters. The lung W/D ratio was significantly increased in the LVT group versus Control group (P<0.01), and was further elevated in HVT group (P<0.001, **Figure 1B**). Besides, lung ultrasound examination (**Figure 1C**) revealed preserved lung contours and A-lines in both Control and LVT groups, whereas the HVT group displayed blurred lung contours, disappearance of A-lines, and extensive confluent B-lines (rocket-tail sign), indicating impaired lung aeration. Moreover, macroscopic examination (**Figure 1D**) showed that HVT-exposed lungs appeared mottled with petechial hemorrhages and fibrinous exudates, whereas Control lungs were uniformly pink and LVT lungs showed only mild congestion. Finally, H&E staining (**Figure 1E**) demonstrated that HVT resulted in severe alveolar structural disruption, intra-alveolar hemorrhage, and extensive inflammatory cell infiltration. Accordingly, the LIS score was markedly higher in the HVT than LVT group (11.5 ± 2.7 vs. 6.5 ± 1.9, P<0.01), and both ventilation groups exhibited higher LIS values than the Control group (2.7 ± 1.4, P<0.05 vs. Control, **Figure 1F**). Western blot analysis of endothelial barrier-associated proteins revealed that VE-cadherin (VE-cad) expression was significantly reduced at all examined time points in both ventilated groups compared with the Control group (all P<0.001). Moreover, VE-cad levels were significantly lower in the HVT group than in the LVT group at P1, P3, and P7 (all P<0.01). Meanwhile, PECAM-1 expression in HVT group was decreased at P0 and P1 but subsequently increased at P3 and P7 compared with Control animals. A similar temporal pattern was observed in LVT group, with a significant difference detected at P1 (P<0.01).

**Figure 1:**
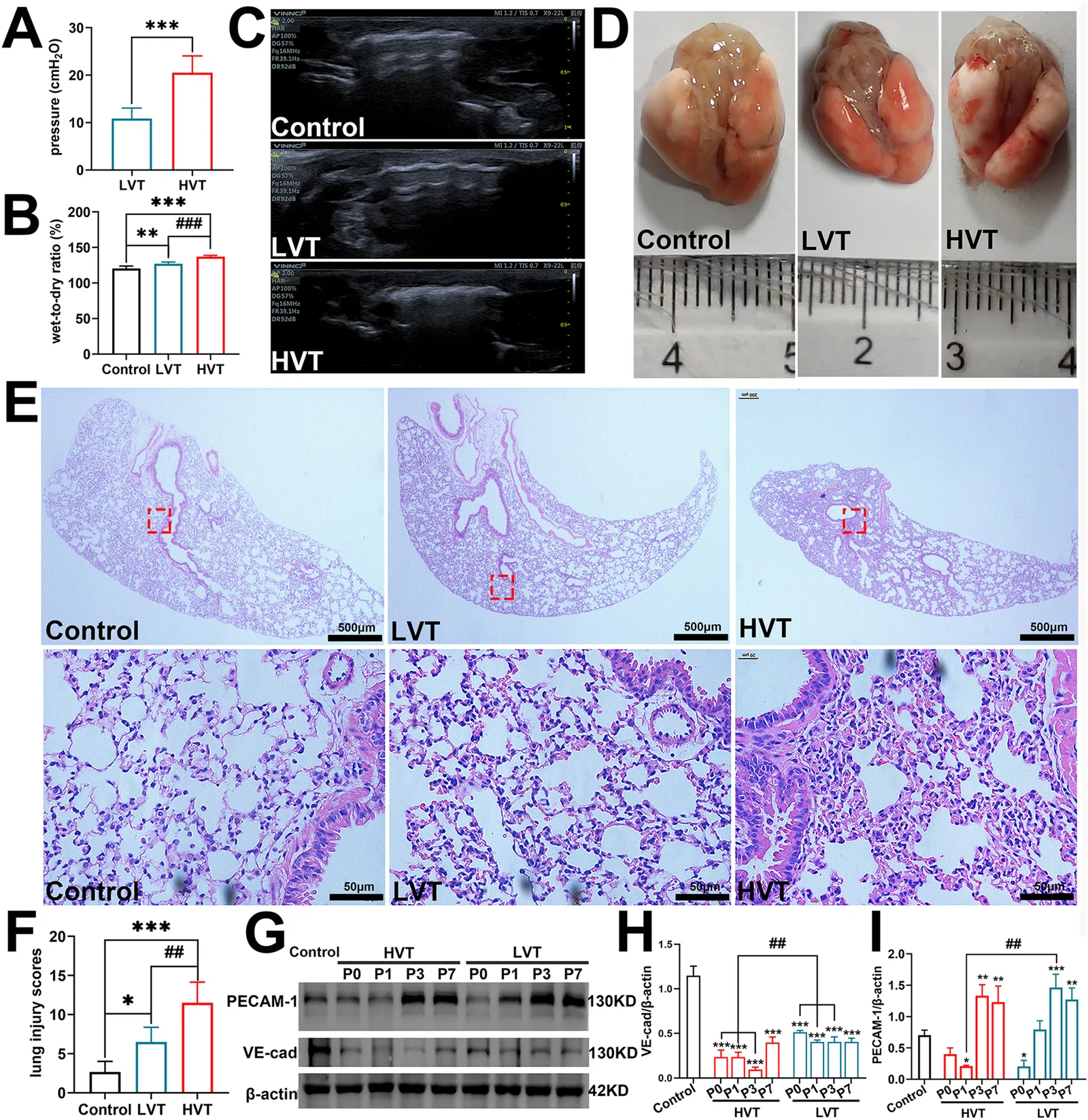
High tidal volume ventilation (HVT) induces acute lung injury in mice. Mice were subjected to Control (intubation without ventilation), LVT (8 mL/kg) or HVT (30 mL/kg) ventilation, lung tissues and ultrasound images were collected at P0 (2 h post ventilation), P1 (1 day post-ventilation), P3 (3 days post-ventilation) and P7 (7 days post-ventilation) for the following analyses: **(A)** Airway plateau pressure during mechanical ventilation in LVT and HVT groups; **(B)** Lung wet-to-dry weight ratio at P1; **(C)** Representative lung ultrasound images at P1; **(D)** Gross morphological appearance of lungs at P1; **(E)** Hematoxylin and eosin (H&E) staining of lung sections (scale bars: 500 μm for low magnification and 50 μm for high magnification); **(F)** Lung injury scores assessed based on H&E staining; **(G–I)** Representative western blots for PECAM-1 and VE-cadherin (VE-cad) in lung tissues at indicated time points after ventilation, with β-actin as the loading control; **(H, I)** Densitometric quantification of PECAM-1 and VE-cad expression, respectively. Unpaired two-tailed Student’s t-test was used for **(A)**, and one-way ANOVA followed by Sidak’s multiple comparison test was used for **(B, F, H, I)**. For **(A–F)**, n=6 mice per group; for **(G–I)**, n=3 mice per group per each time point. *P<0.05, **P<0.01, ***P<0.001 vs. Control group; ^##^ P<0.01, ^###^P<0.001 vs. LVT group. High tidal volume ventilation; LVT, Low tidal volume ventilation; PECAM-1, Platelet endothelial cell adhesion molecule-1; VE-cadherin, Vascular endothelial cadherin.

Collectively, these findings demonstrate that HVT, but not LVT, induces severe acute lung injury characterized by profound physiological impairment, extensive structural damage, and disruption of endothelial barrier integrity.

### HVT aggravates pulmonary macrophage infiltration, M1/M2 polarization imbalance, and IM/AM subset redistribution

To investigate whether differential ventilation strategies affect pulmonary macrophage responses, we first examined inflammation- and polarization-associated proteins. Western blotting (**Figure 2A**) showed that CD11b levels significantly increased in the HVT group at P0, P1, and P3 (each P<0.05), whereas only transient elevation was observed in the LVT group at P0 (P<0.05). Furthermore, CD11b levels were significantly higher in the HVT group than in the LVT group at P1 and P3 (both P<0.01, **Figure 2B**), indicating sustained macrophage accumulation following HVT ventilation. Regarding polarization markers, HVT induced a sustained elevation of M1-associated marker CD86 from P0 to P7, whereas LVT only caused a transient increase at P0 and P1, resulting in significantly higher CD86 levels in the HVT group at P3 and P7 (all P<0.01, **Figure 2C**). Conversely, expression of the M2-associated marker CD204 was significantly reduced in the HVT group compared with Control animals at P1 and P3 (both P<0.001) and compared with the LVT group at P1, P3, and P7 (each P<0.05, **Figure 2D**). Next, RT-PCR confirmed that gene expression levels of M1 markers, including CD86, CD16, IL-6 and TNF-α, were significantly elevated in the HVT group at multiple time points compared with the Control group (all P<0.05), whereas no significant changes were observed in the LVT group (all P>0.05). Moreover, these M1-associated genes were expressed at significantly higher levels in the HVT group than in the LVT group (P<0.05, **Figure 2E**). In contrast, HVT exerted limited effects on M2-associated genes. Although IL-10 expression was increased at P1 and P7 (both P<0.01) and ARG-1 expression was elevated at P1 (P<0.05), YM-1 expression remained unchanged (P>0.05), while CD204 expression was decreased at P1 and P3 (P<0.05). Notably, M2-associated gene expression was significantly higher in the LVT group than in the HVT group (all P<0.05, **Figure 2F**), indicating that HVT predominantly induces M1-associated inflammatory activation while failed to promote M2 polarization.

**Figure 2:**
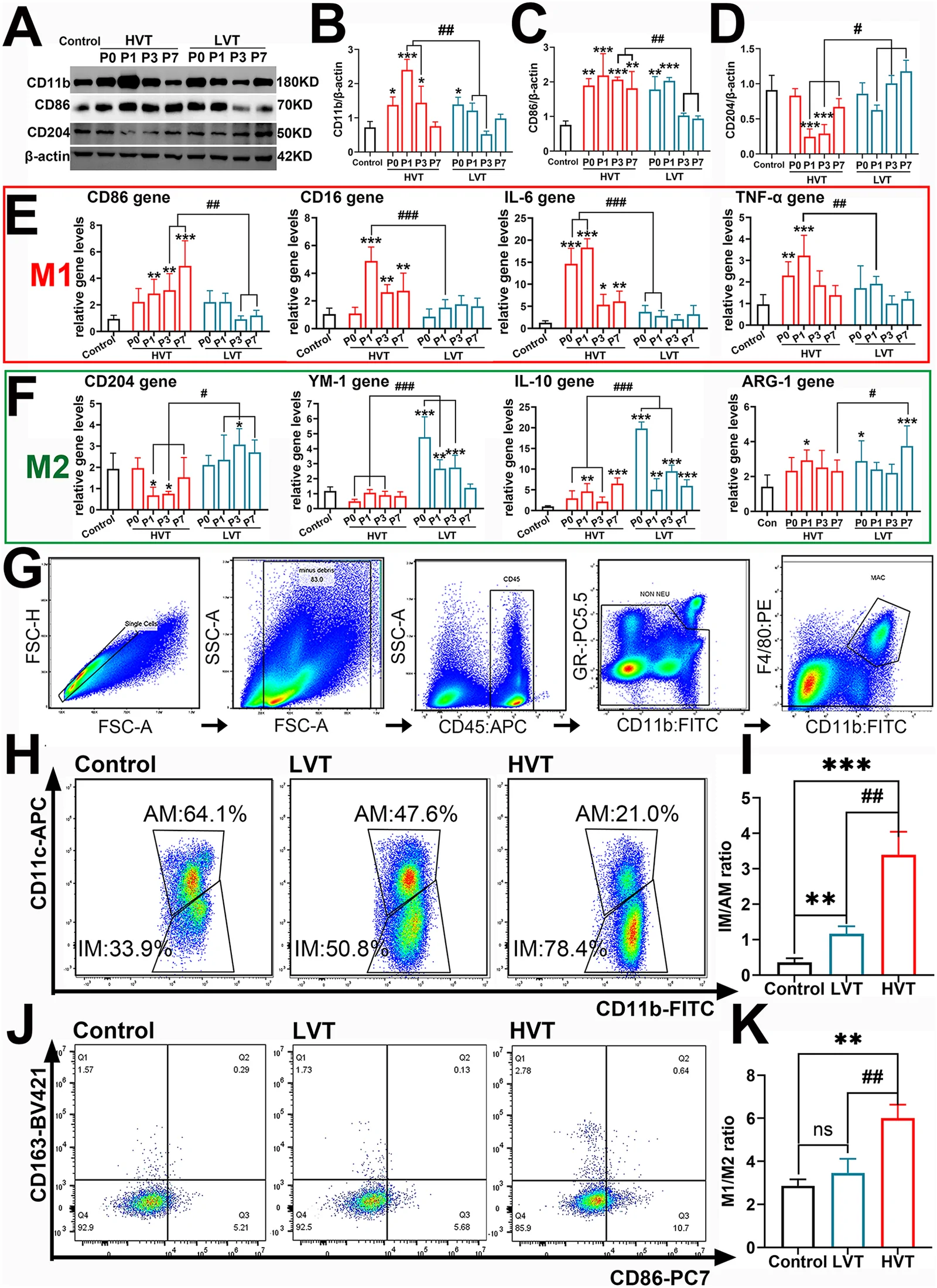
HVT drives macrophage accumulation and phenotypic imbalance in the injured lung. Lung tissues were collected from mice subjected to Control, LVT or HVT ventilation at P0, P1, P3, and P7 for further experiments, including: **(A)** Representative western blots for CD11b, CD86 and CD204 in lung tissues; with β-actin as the loading control; **(B–D)** Densitometric quantification of CD11b, CD86, and CD204 expression, respectively; **(E, F)** Relative mRNA levels of M1-like pro-inflammatory markers (CD86, CD16, IL-6, and TNF-α) and M2-like anti-inflammatory markers (CD204, YM-1, IL-10, and ARG-1) in lung tissues detected by RT-PCR; with β-actin as the loading control; **(G)** Gating strategy for flow cytometry analysis of CD45^+^CD11b^+^F4/80^+^ macrophages; **(H, I)** Representative flow cytometry plots and quantification of AMs (CD11c^+^) and IMs (CD11c^-^); **(J, K)** Representative flow cytometry plots and quantification of M1 (CD86^+^) and M2 (CD163^+^) macrophage subsets. One-way ANOVA followed by Sidak’s multiple comparison test **(B–F, I, K)** was performed. For **(A–D)** n=3 mice per group per each time point; for **(E–K)**, n=6 mice per group per each time point *P<0.05, **P<0.01, ***P<0.001 vs. Control group; ^#^P<0.05, ^##^P<0.01, ^###^P<0.001 vs. LVT group. IL-6, interleukin-6; TNF-α, tumor necrosis factor-α; ARG-1, Arginase-1; YM-1, Chitinase-like protein 3; IL-10, interleukin-10; AMs, Alveolar macrophages; IMs, Interstitial macrophages.

Flow cytometry was subsequently performed to characterize macrophage subsets and polarization status (**Figure 2G**). Lung macrophages were identified as CD45^+^/CD11b^+^/F4/80^+^ cells and further classified into CD11c^+^ (AMs) and CD11c^-^ (IMs). The IM/AM ratio was significantly increased in the HVT group compared with both the Control and LVT groups (P<0.01; **Figures 2H, I**), demonstrating enhanced recruitment and accumulation of inflammatory IMs. Furthermore, macrophage polarization was evaluated using CD86 and CD163 as markers of M1-like (CD86^+^CD163^-^) and M2-like (CD86^-^CD163^+^) macrophages. The M1/M2 ratio was markedly elevated in the HVT group compared with both Control and LVT groups (P<0.01; **Figures 2J, K**), confirming that HVT drives macrophage polarization toward a pro-inflammatory M1-dominant phenotype.

Overall, these results demonstrate that HVT, compared with LVT, induces sustained pulmonary macrophage recruitment, promotes M1/M2 polarization imbalance, and enhances inflammatory IM accumulation in lungs, suggesting that macrophage remodeling represents a critical component of VILI pathogenesis.

### HVT upregulates BMPRIB expression in macrophages

We next investigated whether mechanical ventilation alters the expression pattern of BMP receptors, including BMPRIA, BMPRIB, and BMPRII (**Figure 3A**). Western blot analysis revealed that BMPRII expression was significantly increased in the LVT group at P1 and P3 compared with the Control group (both P<0.05); while HVT ventilation resulted in a significant reduction of BMPRII, which was lower than Control group at P1, and lower than the LVT group at P0, P1, and P3 (each P<0.05, **Figure 3B**). Unlike BMPRII, BMPRIA expression remained unchanged among the three groups throughout the observation period (each P>0.05, **Figure 3C**), suggesting that BMPRIA was not substantially affected by different ventilation strategies.

**Figure 3:**
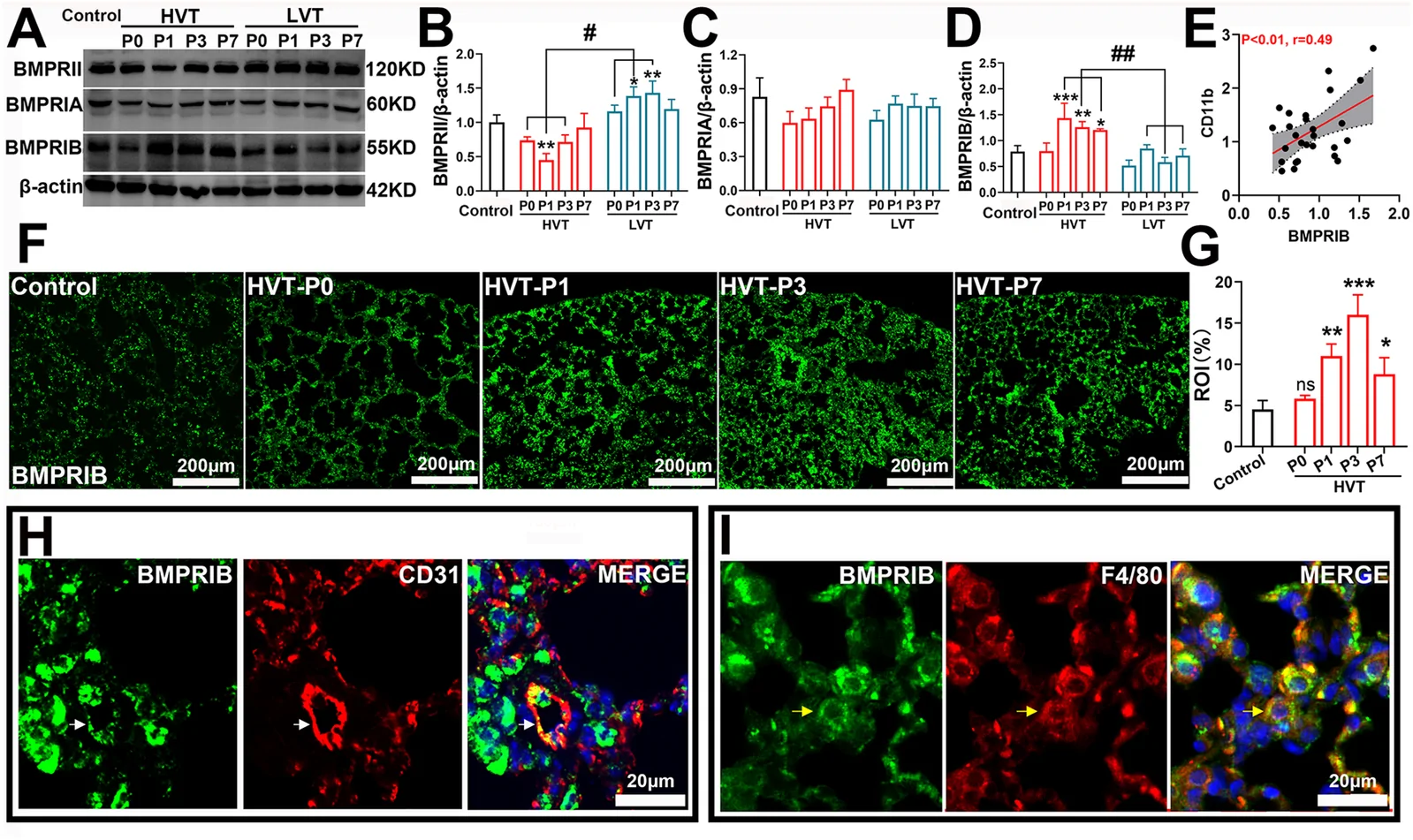
HVT alters BMPR expression and cellular localization. Lung tissues were collected from mice subjected to control, LVT or HVT ventilation at P0, P1, P3, and P7 for further experiments, including: **(A)** Representative western blots for BMPRII, BMPRIA and BMPRIB in lung tissues, with β-actin as the loading control; **(B–D)** Densitometric quantification of BMPRII, BMPRIA, and BMPRIB expression, respectively; **(E)** Pearson correlation analysis between BMPRIB and CD11b expression levels in lung tissues from Control, LVT and HVT groups across all indicated time points; **(F)** Representative immunofluorescence images of BMPRIB expression in lung tissues from Control and HVT groups at P0, P1, P3, and P7 (scale bar=200 μm); **(G)** Quantitative analysis of BMPRIB-positive ROI percentage; **(H, I)** Co-localization of BMPRIB (green) with endothelial cell marker CD31 (red) and macrophage marker F4/80 (red) in HVT-P3 lung tissues (scale bar=50 μm). One-way ANOVA followed by Sidak’s multiple comparison test was used for **(B–D, G)** and Pearson correlation analysis was used for **(E)**. n=3 mice per group per each time point. *P<0.05, **P<0.01, ***P<0.001 vs. Control group; ^#^P<0.05, ^##^P<0.01 vs. LVT group. BMPR, bone morphogenetic protein receptor; ROI, region of interest. The symbol of ns means no significance.

Notably, BMPRIB displayed a distinct expression pattern. Both western blotting and immunofluorescence staining demonstrated that BMPRIB expression was significantly increased in the HVT group compared with both Control and LVT groups at P1, P3, and P7 (all P<0.05, **Figures 3D, F, G**). Correlation analysis further revealed a positive correlation between BMPRIB expression and CD11b levels (P<0.01, r=0.49, **Figure 3E**), suggesting an association between BMPRIB upregulation and macrophage accumulation. To further identify the cellular source of BMPRIB in injured lungs, immunofluorescence co-staining was performed. BMPRIB exhibited partial colocalization with the endothelial cell marker CD31 (**Figure 3H**), but showed extensive colocalization with the macrophage marker F4/80 (**Figure 3I**), indicating that macrophages constitute a major population expressing BMPRIB.

Taken together, these findings identified BMPRIB as a macrophage-associated receptor selectively induced by HVT, suggesting that BMPRIB may participate in macrophage-mediated inflammatory responses during VILI.

### Exogenous BMP4 exacerbates HVT-induced lung injury and macrophage infiltration, which is blocked by BMPRI antagonist LDN193189

Given the selective upregulation of BMPIB expression following HVT ventilation, we next examined whether enhanced BMP signaling contributes to VILI progression. Mice subjected to HVT ventilation received exogenous BMP4 treatment with or without the BMPRI antagonist LDN193189. Compared with the HVT+PBS group, BMP4 administration markedly aggravated lung structural damage, as evidenced by increased alveolar disruption and prominent alveolar wall thickening on H&E staining (**Figure 4A**), which resulted in significantly increased LIS value (P<0.01, **Figure 4B**). Immunofluorescence staining further demonstrated that BMP4 treatment significantly increased the number of F4/80-positive cells in lung tissues (P<0.001, **Figures 4C, D**). Consistently, flow cytometric analysis revealed a significant elevation of the IM/AM ratio following BMP4 administration (P<0.01, **Figures 4E, F**), indicating enhanced recruitment and accumulation of inflammatory IMs.

**Figure 4:**
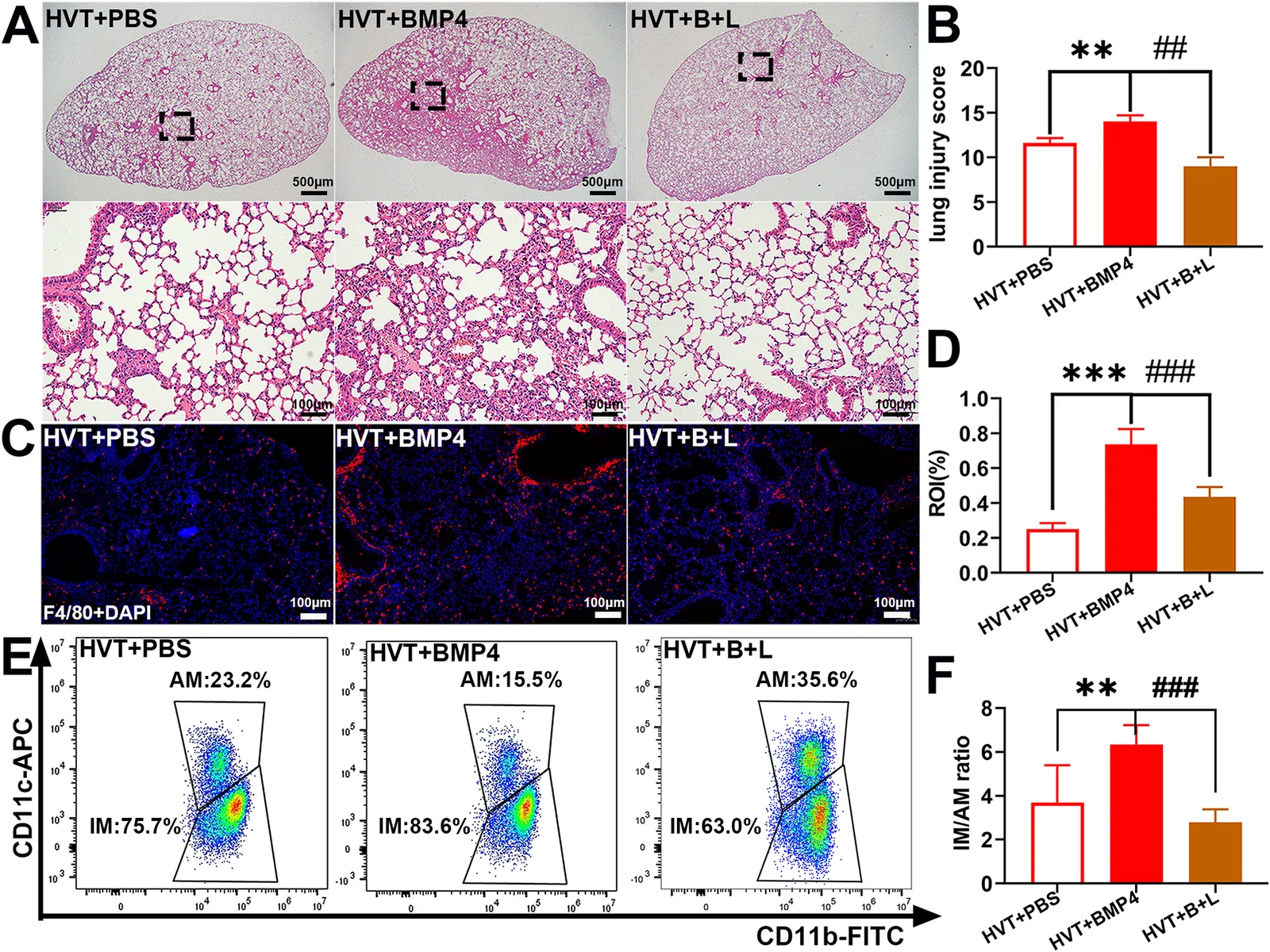
BMP4 treatment aggravates HVT-induced lung injury, reversed by BMP type I receptor blockade. Mice were subjected to HVT and treated with PBS (HVT+PBS), BMP4 (200 ng, HVT+BMP4), or BMP4 plus LDN-193189 (10 μg, a BMP type I receptor inhibitor; HVT+B+L). Lung tissues were collected at P3 for further experiments, including: **(A)** H&E staining of lung sections (scale bars=500 μm for low magnification and 100 μm for high magnification); **(B)** Quantitative lung injury scores based on H&E-stained sections; **(C)** Representative immunofluorescence images of F4/80 (red) with DAPI counterstain (blue) in lung tissues (scale bar=100 μm); **(D)** Quantification of F4/80-positive area as percentage of ROI; **(E, F)** Representative flow cytometry plots and quantification of AMs and IMs. One-way ANOVA followed by Sidak’s multiple comparison test was used for **(B, D, F)**. For **(A, C, E)**, n=6 mice per group. **P<0.01, ***P<0.001 vs. HVT+PBS group; ^##^P<0.01, ^###^P<0.001 vs. HVT+BMP4 group.

In contrast, pharmacological blockade of BMPRI signaling with LDN193189 substantially reversed these BMP4-induced pathological changes. Compared with the HVT+BMP4 group, combined BMP4 and LDN193189 treatment statistically reduced LIS values, decreased F4/80-positive macrophage accumulation, and restored the IM/AM ratio toward baseline levels (all P<0.01 HVT+B+L vs. HVT+BMP4 group, **Figures 4A–F**).

Together, these findings demonstrate that excessive BMP4 signaling aggravates HVT-induced lung injury by promoting macrophage recruitment and inflammatory accumulation, whereas BMPRI inhibition effectively mitigates these detrimental effects.

### Transcriptomic and functional analyses identify BMP4-induced MAPK/NF-κB activation and M1 polarization, reversed by LDN193189

To elucidate the molecular mechanisms underlying BMP4-mediated aggravation of VILI and the protective effects of LDN193189, transcriptomic analysis was performed on lung tissues from the three experimental groups (**Figures 5A–F**). BMP4 treatment upregulated a panel of pro-inflammatory genes, including chemokines (Cxcl2), macrophage-associated genes (Chi3l1, Marco), and stress-associated genes (Hspa1a, Hspa1b); while downregulating genes involved in lipid metabolism and anti-inflammatory signaling (**Figure 5A**). Conversely, LDN193189 co-treatment largely reversed this transcriptional profile by reducing stress-associated genes (Hspa1a, Hspa1b, and Mapk15) and chemokines (Ccl2, Ccl17, Cxcl5, Cxcl2), while upregulated genes linked to ion transport (Atp7a) and fibrinolysis (Serpine1) (**Figure 5D**). Functional enrichment analysis further supported this antagonism. In detail, GO analysis revealed that BMP4-treated lungs were enriched in inflammatory response, innate immunity, cell adhesion, and apoptosis. In contrast, LDN193189 treatment shifted the profile toward signal transduction and negative regulation of apoptosis (**Figures 5B, E**). KEGG pathway analysis revealed that BMP4 activated multiple inflammatory signaling pathways, including chemokine, IL-17, TNF, and MAPK signaling, all of which were reversed by LDN193189 (**Figures 5C, F**). Together, these data indicate that BMP4 promotes a pro-inflammatory gene expression program associated with immune cell recruitment and inflammatory activation, which is effectively counteracted by BMPRI blockade.

**Figure 5:**
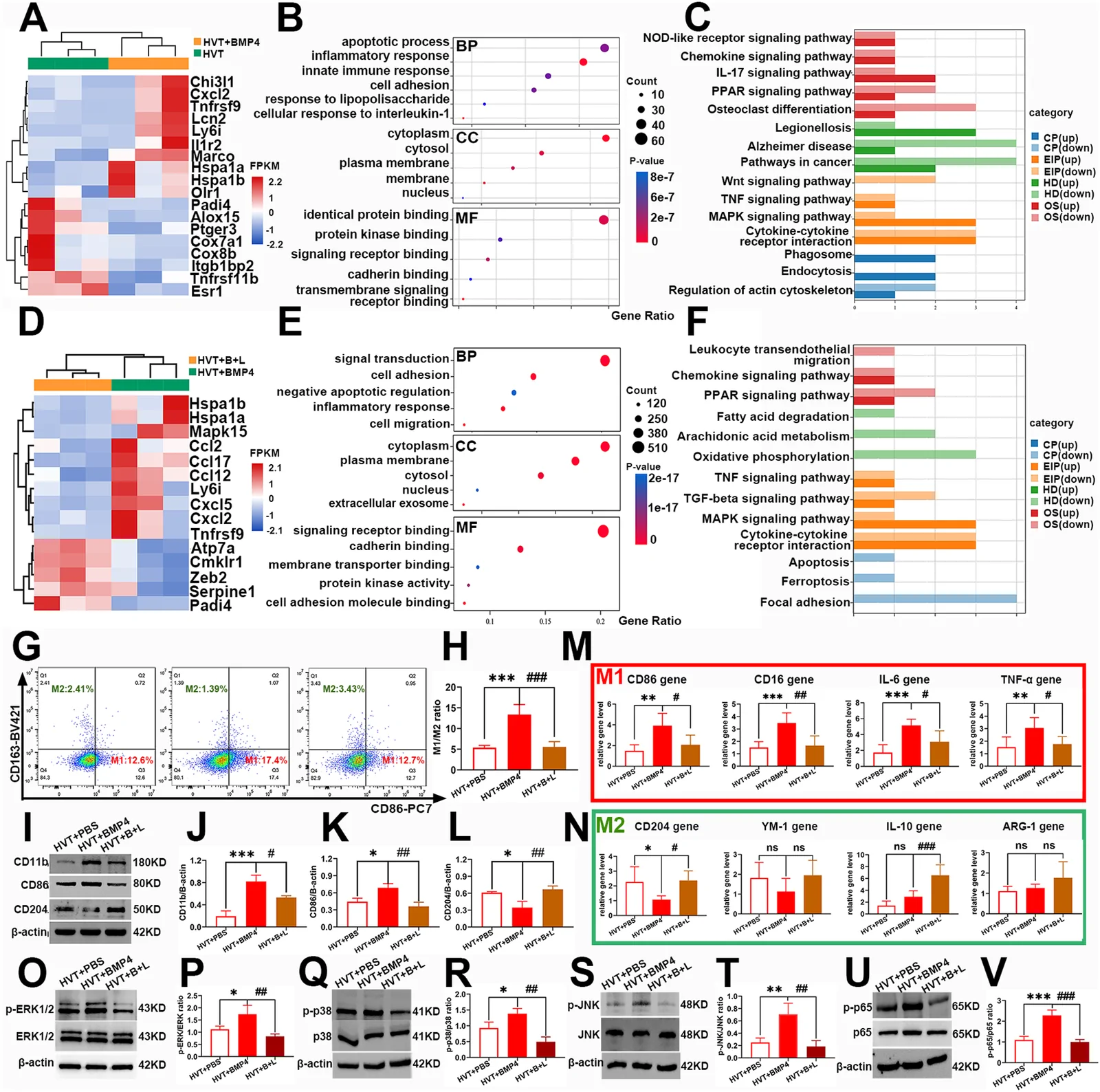
BMP4 induces MAPK/NF-κB activation and M1 polarization via BMP type I receptor in VILI. Lung tissues were collected from mice subjected to HVT and treated with PBS (HVT+PBS), BMP4 (HVT+BMP4), or BMP4+LDN193189 (HVT+B+L) at P3 for further experiments, including: **(A–F)** Heatmap of differentially expressed genes (DEGs), Gene Oncology (GO) enrichment analysis, and Kyoto Encyclopedia of Genes and Genomes (KEGG) pathway enrichment analysis in lung tissues between HVT+PBS and HVT+BMP4 groups, and between HVT+BMP4 and H+B+L groups. **(G, H)** Representative flow cytometry plots and quantification of M1 (CD86^+^) to M2 (CD163^+^) macrophage ratio; **(I–L)** Representative western blotting for CD11b, CD86 and CD204 in lung tissues, with β-actin as the loading control; **(M, N)** Relative mRNA levels of M1-like pro-inflammatory markers (CD86, CD16, IL-6 and TNF-α) and M2-like anti-inflammatory markers (CD204, YM-1, IL-10 and ARG-1) in lung tissues detected by RT-PCR, with β-actin as the loading control; **(O,V)** Representative western blots and densitometric quantification for p-ERK/ERK, p-p38/p38, p-JNK/JNK, and p-p65/p65 ratios in lung tissues. One-way ANOVA followed by Sidak’s multiple comparison test was used for **(H, J–L, M, N, P, R, T, V)**. For **(A–F, I–L, O–V)**, n=3 per group. For **(G, H, M, N)**, n=6 per group. *P<0.05, **P<0.01, ***P<0.001 vs. Control group; ^#^P<0.05, ^##^P<0.01, ^###^P<0.001 vs. LVT group. DEGs: Differentially expressed genes; GO, Gene Ontology; KEGG, Kyoto Encyclopedia of Genes and Genomes; ERK, Extracellular signal-regulated kinase; JNK, c-Jun N-terminal kinase.

Consistent with these molecular findings, flow cytometry confirmed that BMP4 treatment significantly increased the M1/M2 ratio, whereas LDN193189 treatment restored this imbalance (both P<0.001, **Figures 5G, H**). Western blotting and RT-PCR corroborated this polarization shift: BMP4 upregulate M1 markers (CD86, CD16, IL-6, TNF-α) and suppressed M2 marker CD204 at both protein and mRNA levels; These alterations were reversed by LDN193189 treatment, which additionally enhanced M2-associated IL-10 gene expression (all P<0.05, **Figures 5I–N**), suggesting that BMPRI inhibition suppresses BMP4-induced M1 pro-inflammatory polarization while partially restoring an anti-inflammatory M2 phenotype.

Given that MAPK signaling was significantly enriched in BMP4-treated tissues, and considering its established interaction to NF-κB mediated inflammatory response (20, 21), we next examined both pathways by western blotting. BMP4 administration led to a marked increase in the phosphorylation of ERK, JNK, p38, and the NF-κB subunit p65. These phosphorylation events were all attenuated by LDN193189 treatment (all P< 0.05, **Figures 5O–R**), indicating that BMP4 activates both MAPK and NF-κB signaling through a BMPRI-dependent manner.

In summary, these findings demonstrate that BMP4 exacerbates HVT-induced lung injury and drives M1 polarization via activation of MAPK and NF-κB signaling, whereas BMPRI inhibition reverses these pro-inflammatory effects and restores macrophage homeostasis.

### BMPRIB drives macrophage chemotaxis and M1 polarization *in vitro*

To further determine whether BMPRIB directly regulates macrophage inflammatory behavior, BMPRIB expression was manipulated in RAW264.7 macrophages using loss-and gain-of-function approaches. First, the efficiency of BMPRIB knockdown and overexpression were validated. Western blotting and RT-PCR confirmed that transfection with si-SCR or empty vector did not alter BMPRIB expression at protein and mRNA levels compared with transfection reagent control (Mock) (P>0.05). In contrast, si-BMPRIB markedly reduced, whereas oe-BMPRIB increased BMPRIB levels (all P<0.01, **Figures 6A–E**). Immunofluorescence staining further confirmed these alterations, showing decreased BMPRIB fluorescence following si-BMPRIB and enhanced fluorescence after oe-BMPRIB transfection (both P<0.05, **Figures 6G–I**).

**Figure 6:**
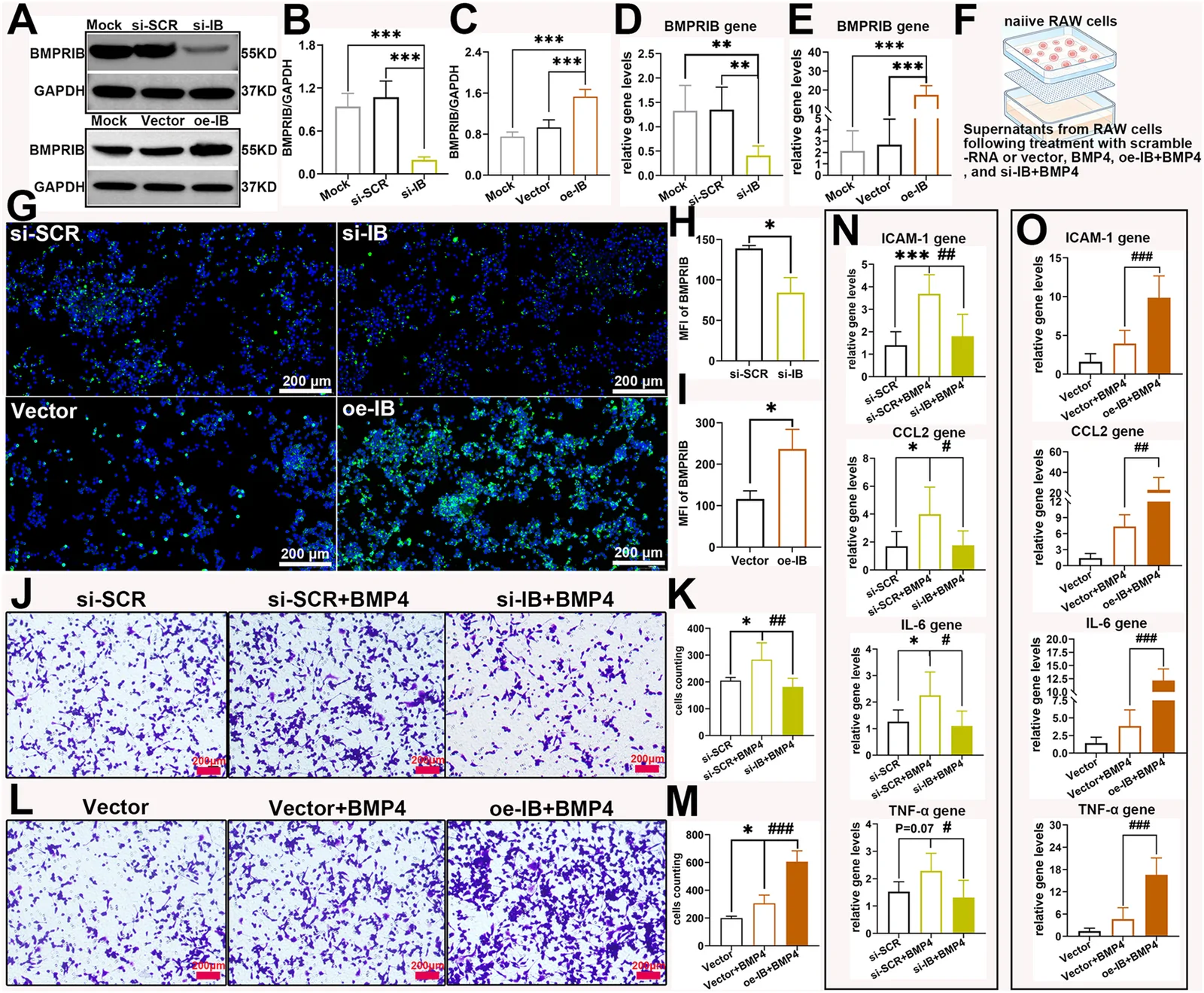
BMPRIB mediates BMP4-induced macrophage polarization, migration and inflammation *in vitro.* RAW264.7 macrophages were transfected with Mock (transfection reagent only), si-SCR (scrambled siRNA), Vector (plasmid), si-IB (knockdown of BMPRIB), or oe-IB (overexpression of BMPRIB) for 48 h, and then treated with 100 ng/mL BMP4 for 48 h. Following experiments included: **(A–C)** Representative western blots and densitometric quantification of BMPRIB expression, with β-actin as the loading control; **(D, E)** Relative mRNA levels of BMPRIB detected by RT-PCR following same transfections, with β-actin as the loading control; **(F)** Schematic diagram of Transwell migration assay; **(G–I)** Representative immunofluorescence images and quantification of BMPRIB expression (scale bar=200 μm); **(J–M)** Representative crystal violet staining images and quantification of migrated macrophages (scale bar = 200 μm); **(N, O)** Relative mRNA levels of ICAM-1, CCL-2, IL-6 and TNF-α detected by RT-PCR. One-way ANOVA followed by Sidak’s multiple comparison test was used for **(B–E, K, M–O)**, and unpaired two-tailed Student’s t-test was used for **(H, I)**. For **(A–C)**, n=3 independent experiments for each group. For **(D, E, G–I, J–O)**, n=6 independent experiments per each group. *P<0.05, **P<0.01, and ***P<0.001 vs. Mock or si-SCR or Vector group; ^#^P<0.05, ^##^P<0.01, ^###^P<0.001 vs. si-SCR+BMP4 or Vector+BMP4 group. RAW264.7, Murine macrophage cell line; NC, si-SCR (scrambled si-RNA); oe-IB, BMPRIB overexpression; si-IB, BMPRIB small interfering RNA; MFI, mean fluorescence intensity; CCL2, C-C motif chemokine ligand 2; ICAM-1, intercellular adhesion molecule-1.

We next investigated the functional impact of BMPRIB on macrophage migration using Transwell assays (**Figures 6F, J–M**). Conditioned media collected from BMP4-stimulated RAW 264.7 macrophages significantly enhanced the migration capacity of naïve RAW264.7 cells transfected with si-SCR or empty vector (both P<0.05). Importantly, BMPRIB expression substantially influenced this chemotactic response. Knockdown of BMPRIB significantly attenuated BMP4-induced macrophage migration compared with the si-SCR+BMP4 group (P<0.01), whereas BMPRIB overexpression further enhanced BMP4-induced migratory activity (P<0.001; BMP4+oe-BMPRIB vs. Vector+BMP4 group).

To further characterize the inflammatory phenotype regulated by BMPRIB, we examined the expression of chemokines and cytokines associated with macrophage activation by RT-PCR. BMP4 stimulation significantly upregulated ICAM-1, CCL2, and IL-6 in si-SCR or vector transfected cells (both P<0.05), whereas TNF-α showed a modest upward trend that did not reach statistical significance (P = 0.07, **Figures 6N, O**). Modulation of BMPRIB expression profoundly altered this response: knockdown of BMPRIB significantly reduced the levels of all four factors (each P<0.05, **Figure 6N**), whereas overexpression further enhanced the expression of these inflammatory mediators (all P<0.01, **Figure 6O**).

Collectively, these findings demonstrate that BMPRIB functions as a critical regulator of macrophage inflammatory activation, promoting chemokine/cytokine production, and enhancing macrophage migratory capacity.

### p38 MAPK mediates BMP4-induced inflammatory and chemotactic signaling in a BMPRIB-dependent manner

Given that HVT selectively upregulated BMPRIB expression in lung macrophages (**Figure 3**) and that exogenous BMP4 activated MAPK signaling pathways *in vivo* (**Figure 5**), we next investigated whether BMPRIB mediates BMP4-induced inflammatory responses through specific MAPK cascades. To test this, we first performed western blotting and found that BMP4 treatment markedly increased the phosphorylation levels of p38, ERK, JNK and NF-κB p65 in cells treated with si-SCR or empty vector (all P<0.05). BMPRIB overexpression further enhanced BMP4-induced phosphorylation of p38 (P<0.01), ERK (P<0.05) and p65 (P<0.001), whereas it did not significantly affect JNK phosphorylation (P>0.05). Conversely, BMPRIB knockdown attenuated BMP4-induced phosphorylation of p38 (P<0.001) and p65 (P<0.01), while phosphorylation of ERK and JNK remained unchanged (both P>0.05; **Figures 7A–J**). Notably, the phosphorylation levels of p38 and p65 showed a strong positive correlation (P<0.01, r=0.80, **Figure 7K**), whereas no significant correlation was observed for ERK or JNK with p65 (both P > 0.05, **Figures 7L, M**). These results suggested that p38 MAPK, rather than ERK or JNK, represents a specific downstream signaling mediator linking BMPRIB activation to NF-κB pathway activation.

**Figure 7:**
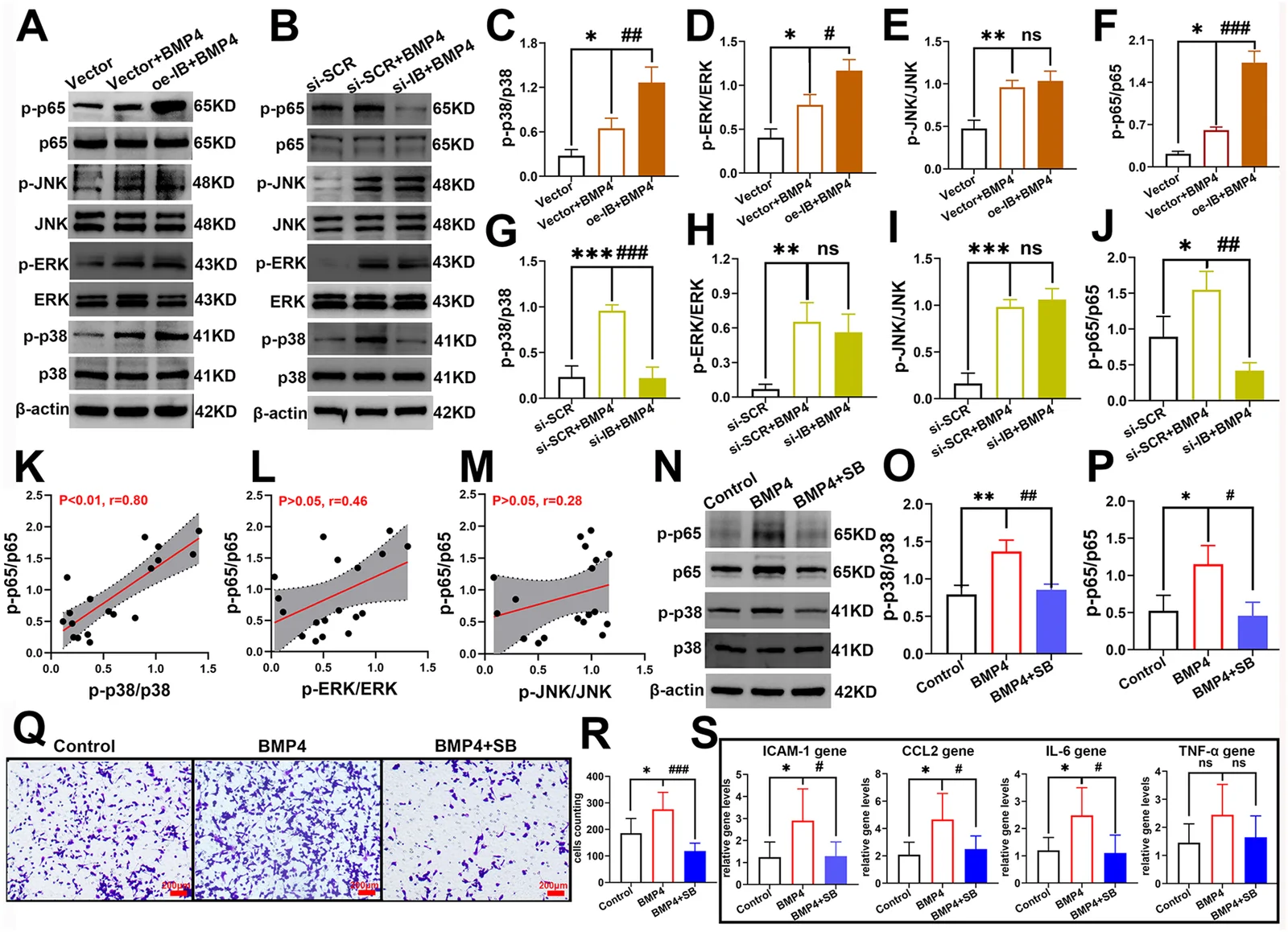
BMPRIB mediates BMP4-induced macrophage activation via p38 MAPK and NF-κB signaling pathway. RAW264.7 macrophages were subjected to NC, oe-IB or si-IB, and then treated with BMP4 for further experiments, including: **(A, B)** Representative western blots for phosphorylated and total p38, ERK, JNK and p65 in cells transfected with oe-IB or si-IB with or without BMP4 treatment; **(C–J)** Densitometric quantification of p-p38/p38, p-ERK/ERK, p-JNK/JNK and p-p65/p65 ratios in overexpression and knockdown groups; **(K–M)** Pearson correlation analysis between p-p65/p65 and p-p38/p38, p-ERK/ERK and p-JNK/JNK protein levels. Then, RAW264.7 macrophages were pretreated with 1 μmol/L SB202190, followed by stimulation of 100 ng/mL BMP4 for 48 h. The tests included: **(N–P)** Representative western blots and densitometric quantification of p-p38/p38 and p-p65/p65 ratios; **(Q, R)** Representative crystal violet staining images and quantification of migrated macrophages after SB203580 treatment (scale bar=200 μm); **(S)** Relative mRNA levels of CCL2, IL-6 and TNF-α in each group detected by RT-PCR. One-way ANOVA followed by Sidak’s multiple comparison test was used for **(C–J, O, P, R, S)** and Pearson correlation analysis was used for **(K–M)**. For **(A–P)**, n=3 independent experiments per group. For **(Q–S)**, n=6 independent experiments per group. For **(C–J)**, *P<0.05, **P<0.01, and ***P<0.001 vs. si-SCR or Vector group, ^#^P<0.05, ^##^P<0.01, ^###^P<0.001 vs. si-SCR+BMP4 or Vector+BMP4 group. For **(O,P, R, S)**, *P<0.05, **P<0.01, and ***P<0.001 vs. Control group; ^#^P<0.05, ^##^P<0.01, ^###^P<0.001 vs. BMP4 group. None. The symbol of ns means no significance.

To further validate the functional role of p38, macrophages were treated with SB202190 (a specific p38 inhibitor). Western blotting showed that SB202190 effectively suppressed BMP4-induced phosphorylation of both p38 and p65 (both P<0.05, Figure N-P). Furthermore, Transwell migration assays revealed that SB202190 significantly attenuated the chemotactic activity induced by conditioned media from BMP4-treated RAW264.7 macrophages (P<0.05, **Figures 7Q, R**). Consistently, RT-PCR analysis demonstrated that SB202190 markedly decreased BMP4-induced ICAM-1, CCL2 and IL-6 expression (both P < 0.05), whereas TNF-α expression showed a reduction which did not reach statistical significance (P = 0.27, **Figure 7S**).

Taken together, these results establish that BMP4 activates a BMPRIB-dependent p38 MAPK/NF-κB signaling cascade in macrophages, thereby contributing to macrophage accumulation and inflammatory amplification during VILI.

## Discussion

In this study, we identified a previously unrecognized role of the BMPRIB-p38 MAPK-p65 signaling axis in regulating macrophage-mediated inflammation during VILI. Four major findings were obtained (**Figure 8**). First, compared with LVT, HVT induces more severe lung injury characterized by enhanced macrophage accumulation, M1/M2 polarization imbalance, and a significantly increased IM/AM ratio, suggesting that macrophage subset remodeling represents an important feature of VILI progression (**Figure 8A**). Second, among the BMP receptors examined, BMPRIB is selectively upregulated and largely co-localized with macrophages in HVT lungs, whereas BMPRIA remains unchanged and BMPRII is downregulated, pointing to a macrophage-specific receptor switch that may shift BMP signaling from homeostatic to pro-inflammatory (**Figure 8B**). Third, exogenous BMP4 administration exacerbates HVT-induced lung injury and macrophage accumulation, whereas co-administration of BMPRI blockade-LDN193189-reverses these BMP4-induced effects, supporting a functional contribution of BMP signaling in VILI pathogenesis (**Figure 8C**). Fourth, using complementary gain- and loss-of-function approaches *in vitro*, we confirm that BMPRIB directly regulates BMP4-induced chemokine/cytokine production and macrophage migration, with p38 MAPK/NF-κB serving as a key downstream signaling hub (**Figure 8D**); Collectively, these findings identify the BMP4-BMPRIB-p38 MAPK-p65 axis as a potential therapeutic target for limiting excessive pulmonary inflammation during VILI.

**Figure 8:**
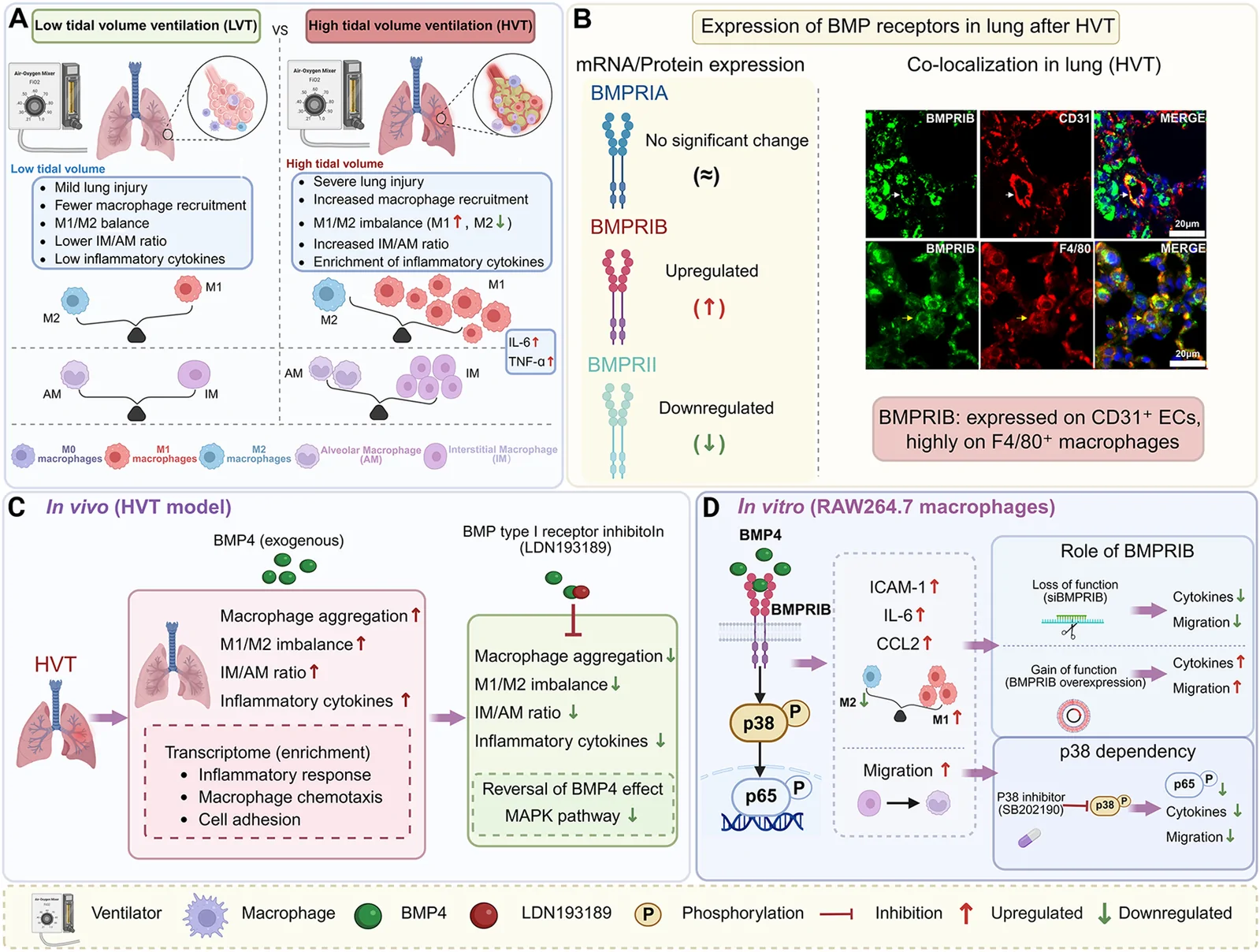
Schematic summary of the mechanism by which BMPRIB-p38 MAPK/NF-κB signaling mediates macrophage inflammatory reprogramming and chemotaxis in VILI. The schematic integrates the major findings of this study: **(A)** HVT, compared with LVT, induces more severe lung injury, macrophage accumulation, M1/M2 imbalance and increased IM/AM ratio; **(B)** Among BMP receptors examined, BMPRIB is selectively upregulated and largely co-localized with macrophages in HVT lungs; **(C)** exogenous BMP4 administration exacerbates HVT-induced lung injury and macrophage accumulation, effects reversed by co-treatment of BMP type I receptor antagonist LDN193189, **(D)** BMPRIB directly mediates BMP4-induced chemokine/cytokine production and macrophage migration via p38 MAPK and NF-κB signaling pathways. Collectively, these findings identify the BMP4-BMPR IB-p38-NF-κB signaling cascade as a critical driver of VILI pathogenesis and a potential therapeutic target for this condition. Image was created in https://BioRender.com.

Clinical evidence regarding optimal intraoperative ventilation strategies remains inconclusive. A landmark study demonstrated that LVT (6 mL/kg) reduces mortality in ARDS patients compared with traditional tidal volumes (12 mL/kg) (22). Furthermore, a Cochrane systematic review extended the potential benefits of LVT to surgical populations, reporting that LVT (below 10 mL/kg) is associated with reduced postoperative pneumonia incidence and decreased requirements for ventilatory support compared with HVT (above 10 mL/kg) (23). However, a large randomized trial reported no significant difference in clinical outcome between LVT (6 mL/kg) and HVT (10 mL/kg) when positive end-expiratory pressure (PEEP) was equalized (24). These inconsistent findings suggest that the biological mechanisms distinguishing protective from injurious ventilation are not fully understood. In the present study, we established a murine ventilation model in which ventilation intensity was characterized by airway plateau pressure using a 22G intravenous catheter sheath as an uncuffed endotracheal tube. The resulting plateau pressures (HVT: 20.5 ± 3.6 cm H_2_O; LVT: 10.8 ± 2.2 cm H_2_O), that closely mirror clinically relevant differences between injurious and protective ventilation models. Under these conditions, HVT induced significantly greater pulmonary injury than LVT, as demonstrated by multiple independent indicators, including gross morphological appearance, ultrasound abnormalities, increased wet/dry ratio, histological destruction, and disruption of endothelial barrier-associated proteins (**Figure 1**). Importantly, although LVT caused mild macrophage activation, HVT triggered persistent macrophage accumulation, M1/M2 imbalance, and increase in the IM/AM ratio (**Figure 2**). Given that IMs are largely composed of monocyte-derived macrophages (MDMs) recruited from the circulation, whereas AMs are tissue-resident and maintain immune homeostasis (25), we propose that excessive macrophage recruitment and subset imbalance represent key mechanisms contributing to VILI pathogenesis. This is consistent with recent observations that macrophage polarization dynamics play a critical role in both progression and resolution of VILI (26). In addition, we noted a biphasic change in PECAM-1 expression following HVT—decreased at early time points but elevated at later stages—which contrasts with the sustained loss of VE-cadherin and may reflect compensatory endothelial remodeling during injury resolution. Although the functional significance of this observation remains to be determined, it highlights the complexity of endothelial barrier regulation in response to injurious ventilation.

BMP4 was selected as the candidate signaling molecule in this study based on its well-established involvement in lung development and various pulmonary diseases (12), as well as its association with inflammatory responses in clinical settings (27). Notably, BMP4 exerts differential effects on macrophage polarization depending on the pathological context: in pulmonary fibrosis, BMP4 suppresses M2 polarization to alleviate fibrotic progression (28), and has also been reported to ameliorate pulmonary fibrosis by repressing NLRP3 inflammasome activation and epithelial-mesenchymal transition (29); whereas tumor-derived BMP4 promotes M2 polarization to facilitate immune evasion (30). Additionally, endothelial cell-derived BMP4 has been shown to promote MDM recruitment and tissue infiltration through disruption of vascular integrity and induction of adhesion molecules (31–33). Beyond its role as an extracellular signal, BMP4 is also produced by macrophages themselves. For instance, Ara et al. observed increased BMP4 mRNA and protein levels in infiltrating MDMs within the spinal cord of experimental autoimmune encephalomyelitis (EAE) mice (34). Similarly, our previous work demonstrated that BMP4^high^ MDMs following spinal cord injury exhibit enhanced tissue-infiltrating capacity and pro-inflammatory M1 polarization (35). All these lines of evidence support the concept that MDMs serve as both producers and responders of BMP4 signaling under pathological conditions, establishing a potential autocrine/paracrine amplification loop that promotes MDM recruitment and pro-inflammatory polarization.

The differential regulation of macrophage polarization by BMP4 across pathological context, as discussed above, raises a fundamental question: whether macrophages are equipped with the relevant receptors and how receptor-specific signaling shapes their functional polarization. Addressing this, a single-cell RNA sequencing study has identified BMPRIB expression in a subset of human monocytes, providing the molecular basis for specific regulation of BMPR on macrophage lineage cells (36). Using RAW264.7 macrophages, Bai et al. showed that BMP9 enhances macrophage recruitment and phagocytic activity through ALK1 signaling (37). Pharmacological studies further support this receptor-centric notion, since blockade of BMPRI has been shown to restore M1/M2 imbalance and alleviate inflammation in ischemia-reperfusion injury (38, 39). Collectively, these findings demonstrate that BMP signaling directly regulates macrophage polarization in a receptor subtype-dependent manner, and that the functional outcome—whether pro-inflammatory M1 or anti-inflammatory M2 polarization—is determined by which receptor is engaged. In line with this framework, we identify BMPRIB as a critical receptor mediating BMP4’s pro-inflammatory effects in VILI. Through both *in-vivo* and *in-vitro* approaches, we showed that HVT specifically enhanced BMPRIB expression on macrophages, enabling BMP4 to drive M1 polarization and macrophage accumulation, with these effects reversed by LDN193189. In RAW264.7 macrophages, genetic manipulation of BMPRIB expression directly altered BMP4-induced macrophage responses. BMPRIB overexpression enhanced macrophage migration and inflammatory mediator production, whereas BMPRIB silencing substantially attenuated these effects, providing direct evidence that BMPRIB is not merely associated with macrophage activation but actively mediates BMP4-driven inflammatory responses. Transcriptomic analysis further supports a mechanistic link between BMP4 signaling and inflammation-related pathways. Therefore, these findings provide direct evidence that BMPRIB as a potential therapeutic target for VILI, suggesting that receptor-specific intervention may offer a more precise therapeutic strategy for controlling macrophage-mediated inflammatory injury.

The downstream signaling pathways through which BMPRIB mediates its effects during VILI remain largely undefined. As a primary signaling arm downstream of BMP receptors, SMADs have been shown to exert pleotropic and even opposing effects on macrophage polarization. For instance, in an *in vitro* stress microenvironment modeled by apoptotic conditioned medium, SMADs signaling, in coordination with PI3K-Akt-mTOR, promote monocyte to M2 macrophage polarization (40). In the meantime, SMAD signaling inhibits IL-4-induced M2 polarization through suppression of STAT6 in the context of bleomycin-induced pulmonary fibrosis (28). These findings indicate that the functional outcome of SMAD signaling in macrophages is largely determined by which intracellular pathways are co-activated. In contrast to the context-dependent nature of SMAD signaling, non-canonical pathways—particularly the MAPK cascade—are more directly linked to macrophage phenotype switching and inflammatory responses. Among these, the p38 MAPK pathway has emerged as a critical signaling node in M1 polarization. For instance, activation of p38 MAPK and NF-κB signaling has been shown to directly promote M1 polarization in macrophages (41), with pharmacological or genetic inhibition shown to reduce pro-inflammatory cytokine production and restore M1/M2 balance in diverse pathological conditions, including diabetes (42), gouty arthritis (43), ulcerative colitis (44), and acute lung injury (45). However, whether BMPRIB engages p38 MAPK to regulate macrophage polarization during VILI has not been investigated. Our study provides multiple lines of evidence establishing p38 MAPK/NF-κB as the predominant downstream effector of BMPRIB during VILI. First, BMPRIB was significantly upregulated in lung macrophages following HVT ventilation. This observation is functionally relevant, as BMP type I receptors, including BMPRIB, have been shown to directly phosphorylate MAPKs (46); moreover, loss-of-function mutations in BMPRIB abrogate both SMAD-dependent and p38 MAPK signaling, indicating that functional BMPRIB is essential for p38 activation (47). Second, transcriptomic analysis revealed that exogenous BMP4 significantly enriched the MAPK signaling pathway in lung tissues, an effect suppressed by LDN193189. Third, gain- and loss-of-function experiments targeting BMPRIB showed that p38 and p65 phosphorylation strictly depended on BMPRIB expression, whereas ERK and JNK phosphorylation did not; correlation analysis demonstrated a strong positive correlation between p38 and p65 phosphorylation (r = 0.80, P < 0.01), but not for ERK or JNK. Finally, pharmacological inhibition of p38 with SB202190 suppressed p38 and p65 phosphorylation, abrogated BMP4-induced chemokine and pro-inflammatory cytokine expression, and blocked macrophage migration *in vitro*. Collectively, these findings establish the p38–NF-κB axis as the predominant non-canonical pathway downstream of BMPRIB through which BMP4 drives M1 polarization and macrophage accumulation during VILI. Future studies will investigate whether this involves altered nuclear translocation or other regulatory mechanisms.

Several limitations of the present study should be acknowledged. First, although we focused on BMPRIB-mediated P38-MAPK activation, the potential contribution of BMPRII downregulation cannot be excluded. Reduced BMPRII levels have been associated with enhanced inflammatory responses in lung injury models (48, 49). Second, LDN193189 is a non-selective BMPRI inhibitor that targets ACVR1, BMPRIA, and BMPRIB. Thus, future studies using BMPRIB-selective antagonists or subtype-specific genetic models are required to dissect the individual contributions of each BMPRI subtype in VILI. Third, the precise role of canonical SMAD signaling in BMP4-mediated macrophage polarization during VILI remains to be fully characterized, and the potential crosstalk between SMAD and non-canonical pathways warrants systematic investigation. Fourth, while RAW264.7 cells are widely used for macrophage signaling studies, future experiments using primary alveolar macrophages or human monocyte-derived macrophages would further strengthen the translational relevance of our findings.

In summary, we demonstrate that HVT-induced BMPRIB upregulation on lung macrophages enables BMP4 to activate p38MAPK and NF-κB signaling, driving M1 polarization, macrophage accumulation and IM/AM imbalance, thereby exacerbating VILI. These findings identify BMPRIB as a critical upstream regulator of macrophage-mediated inflammation in VILI, and provide the first evidence that BMP receptor subtype-specific signaling determines the direction of macrophage polarization in this disease context. From a translational perspective, our findings suggest that targeting BMPRIB—rather than broadly inhibiting BMP signaling—may offer a more precise strategy to mitigate macrophage-driven lung injury. The development of BMPRIB-specific antagonists could be a promising direction for future therapeutic intervention in VILI.

## Data Availability

The names of the repository/repositories and accession number(s) can be found below: OMIX019178 (https://ngdc.cncb.ac.cn/omix/release/OMIX019178). The original Western blotting bands are included in the supplementary files.

## Supplementary material

Supplementary Figure 1 BMP4 concentration-dependent regulation of inflammatory and chemotactic genes in RAW264.7 macrophages. RAW264.7 macrophages were treated with recombinant mouse BMP4 proteins at concentrations of 0, 50, 100 or 200 ng/mL for 48 h. Relative mRNA levels were measured by RT-PCR for **(A)** ICAM-1; **(B)** CD16; **(C)** CD86; **(D)** CXCL2; **(E)** CCL2; **(F)** IL-6; and **(G)** TNF-α. One-way ANOVA followed by Sidak’s multiple comparison test was performed. n = 6 independent experiments per group. *P<0.05, **P<0.01 vs. 0 ng/mL.
