# Supplementary material for: Macrophage BMPRIB mediates p38-dependent inflammatory reprogramming and chemotaxis during ventilator-induced lung injury
Source: Front Immunol. 2026 Oct 6;17:1947415. doi: 10.3389/fimmu.2026.1947415 (PMC13680878; doi:10.3389/fimmu.2026.1947415)
Supplement: Supplementary file 1 [file DataSheet1.pdf]

frontiers

The International Society for the Advancement of Cytometry (ISAC) has highlighted the importance of including comprehensive methodological information to ensure data reproducibility and reliability. In line with this, Frontiers in Immunology now requires authors to submit a checklist for manuscripts that involve flow or mass cytometry. This checklist helps standardize the reporting process, improving the quality and transparency of published data. By doing so, we support scientific progress, making it easier for other researchers to replicate and validate experiments.

This form should be submitted with any manuscripts using flow or mass cytometry.

Sample/specimen/material description

☐ Total blood

☐ PBMCs

✔ Organ digests

Other____

Did the samples suffer any treatment before or after incubation with the antibodies?

☐ Drug ____________________________________

☐ Cell permeabilization ________________________

✔ Dye __cell surface staining____________________

☐ Propidium iodine

☐ Not applicable

Other ____

Instrument and antibodies

Name of the Cytometer___Beckman flow cytometer：CytoFLEX_________________________

Antibodies and targets	Fluorochrome/ Metal	Catalog number/Company
Rat Anti-Mouse CD45 (30-F11)	APC-Cy7	Cat. 557659, BD Pharmingen

Rat Anti-Mouse Ly-6G and Ly-6C (RB6-8C5)	PerCP-Cy5.5	Cat. 552093, BD Pharmingen
Rat Anti-CD11b (M1/70)	FITC	Cat. 557396, BD Pharmingen
Rat Anti-Mouse F4/80 (T45-2342)	PE	Cat. 565410, BD Pharmingen
Rat Anti-Mouse CD86(G L1)	PE-Cy7	Cat.560582, BD Pharmingen
anti-mouse CD163 Anti body	Brilliant Violet 421	Cat.155309, BioLegend
Hamster Anti-Mouse CD 11c(HL3)	APC	Cat.550261, BD Pharmingen
Live/Dead Discriminat or Dyes	Brilliant Violet 510	Cat.564406, BD Pharmingen
Ms CD16/CD32 Pure 2.4G2	/	Cat.553141, BD Pharmingen

Data analyses

1. Name of the software___FLOWJO 10.8___________________
2. Reference gating strategy in the manuscript or supplementary material

Gating strategy in (eg Figure X) ______Figure2________________________
